# Unequal Gain of Equal Resources across Racial Groups

**DOI:** 10.15171/ijhpm.2017.90

**Published:** 2017-08-05

**Authors:** Shervin Assari

**Affiliations:** ^1^ Center for Research on Ethnicity, Culture and Health (CRECH), School of Public Health, University of Michigan, Ann Arbor, MI, USA.; ^2^ Department of Psychiatry, University of Michigan, Ann Arbor, MI, USA.; ^3^ Institute for Healthcare Policy and Innovation (IHPI), University of Michigan, Ann Arbor, MI, USA.

**Keywords:** Racial Health Disparities, Structural Barriers, Racism, Health Policy, Public Policy

## Abstract

The health effects of economic resources (eg, education, employment, and living place) and psychological assets (eg, self-efficacy, perceived control over life, anger control, and emotions) are well-known. This article summarizes the results of a growing body of evidence documenting Blacks’ diminished return, defined as a systematically smaller health gain from economic resources and psychological assets for Blacks in comparison to Whites. Due to structural barriers that Blacks face in their daily lives, the very same resources and assets generate smaller health gain for Blacks compared to Whites. Even in the presence of equal access to resources and assets, such unequal health gain constantly generates a racial health gap between Blacks and Whites in the United States. In this paper, a number of public policies are recommended based on these findings. First and foremost, public policies should not merely focus on equalizing access to resources and assets, but also reduce the societal and structural barriers that hinder Blacks. Policy solutions should aim to reduce various manifestations of structural racism including but not limited to differential pay, residential segregation, lower quality of education, and crime in Black and urban communities. As income was not found to follow the same pattern demonstrated for other resources and assets (ie, income generated similar decline in risk of mortality for Whites and Blacks), policies that enforce equal income and increase minimum wage for marginalized populations are essential. Improving quality of education of youth and employability of young adults will enable Blacks to compete for high paying jobs. Policies that reduce racism and discrimination in the labor market are also needed. Without such policies, it will be very difficult, if not impossible, to eliminate the sustained racial health gap in the United States.

## Economic Resources and Psychological Assets Impact Health

The protective health effects of economic resources (eg, education and employment)^1^ and psychological assets (eg, self-efficacy, perceived control over life, anger control, and emotions)^2-6^ are well-known. Mirowsky and Ross have described the health effects of socio-economic status (SES) as “enduring, consistent, and growing.”^7^ Psychological assets such as emotion regulation, positive emotions, self-efficacy, and sense of control over life are also essential for maintaining health.^8-10^ These resources and assets increase access to material and human resources which collectively enable individuals to avoid risks and minimize their negative consequences as they occur.^11,12^ The aim of this paper is to review and discuss a growing body of literature which documents systematically smaller health effects of SES resources and psychosocial assets for Blacks compared to Whites in the United States. That is minority status bounds the potential gains that follow SES resources and psychological assets, also known as *Blacks’ diminished return*.^13^

State-of-the-art longitudinal studies, such as the Health and Retirement Study (HRS),^14^ the Panel Study of Income Dynamics,^15^ the British Whitehall Study,^16^ the British Cohort Study (BCS),^17^ the French GAZEL cohort,^18^ the Survey of Health, Aging and Retirement in Europe (SHARE),^19^ and Americans’ Changing Lives (ACL) study^20^ have all documented strong protective effects of educational attainment, employment, and income against risk of morbidity^21^ and mortality.^22^ Longitudinal studies have also shown that high levels of psychological assets such as mastery, self-efficacy, perceived control over life, emotion regulation, positive emotions, and anger control are also protective against morbidity and mortality.^23-28^ In fact, there is some evidence suggesting thatthese psychological assets are one of the mechanisms by which high SES protects health.^29,30^ For instance, mastery is shown to mediate the effects of both earlier- and later-life economic hardships on elders’ physical and mental health. High mastery also buffers the impact of economic hardship on health outcomes of older aults.^31^

## Unequal Gain From Equal Resources and Assets

In nearly 20 papers (see Table 1), my colleagues and I have found that in the United States, economic resources and psychological assets systemically result in a smaller health gain for Blacks compared to Whites. These findings hold for a wide range of SES resources (eg, education,^32^ employment,^33^ and neighborhood safety^34^) as well as psychological assets (eg, emotions,^36-38^ anger management,^39^ sleep quality,^40^ self-efficacy,^41^ perceived control over life,^39^ and self-rated health^35^) on health. These differential effects are found for several physical health outcomes such as incident chronic disease,^36,38,40^ all-cause mortality,^37,41,42^ and cause-specific mortality.^43^ These findings are not specific to a particular risk factor or outcome.^47-50^

**Table 1:** Our Findings on Weaker Effects of Psychosocial Factors on Health of Blacks Than Whites

**Author**	**Data Set**	**Longitudinal**	**Follow Up**	**Gender**	**Predictor**	**Outcome**
Assari and Lankarani^32^	ACL	+	25 Years	Men	Education	All-cause mortality
Assari^33^	ACL	+	25 Years	Both	Employment	All-cause mortality
Assari^34^	ACL	+	25 Years	Both	Neighborhood safety	All-cause mortality
Assari and Lankarani^35^	ACL	+	25 Years	Both	SRH	All-cause mortality
Assari et al^36^	ACL	+	25 Years	Both	Depression	Chronic disease
Assari et al^37^	ACL	+	25 Years	Both	Depression	All-cause mortality
Assari and Lankarani^38^	ACL	+	25 Years	Both	Depression	Chronic disease
Assari^39^	ACL	+	25 Years	Both	Anger and hostility	Cause-specific mortality
Assari et al^40^	ACL	+	25 Years	Both	Restless sleep	Chronic disease
Assari^41^	ACL	+	25 Years	Both	Self-efficacy	All-cause mortality
Assari^42^	RAHS	+	3 Years	Both	Sense of control	All-cause mortality
Assari and Burgard^43^	ACL	+	25 Years	Both	Depression	Cause-specific mortality
Assari et al^44^	HRS	+	6 Years	Men	Education	Sleep, BMI, physical activity
Assari^45^	ACL	+	25 Years	Men	Education	Depression
Assari^46^	NSAL	-	-	Men	Education	Suicidal Ideation
Assari^47^	RAHS	-	-	Both	Life purpose	BMI
Assari and Lankarani^48^	RAHS	-	-	Both	Education	Alcohol Use
Assari and Lankarani^49^	NSAL	-	-	Men	Stress	Depression
Assari^50^	NSAL	-	-	Men	Depression	Obesity
Assari et al^51^	FFWS	+	15 Years	Both	Family SES	Obesity
Assari^52^	NSAL	-	-	Men	Income	Depression
Assari^53^	FFWS	+	15 Years	Both	Family SES	SRH
Assari and Lankarani^54^	NSAL	-	-	Both	Obesity	Intention to reduce weight

Abbreviations: SRH‏, Self-rated Health; BMI, body mass index; ACL, Americans’ Changing Lives; SES, socio-economic status; HRS, Health and Retirement Study; RAHS, Religion, Aging, and Health Survey; FFWS, Fragile Families and Child Wellbeing Study; NSAL, National Survey of American Life.

My team and I have generated these findings using the following national longitudinal studies: (1) the ACL study, 1986-2011, a 25-year cohort of 3600+ adults, (2) the Midlife in the United States (MIDUS) study, 1995-2004, a 10-year cohort of 7100+ adults, and (3) the Religion, Aging, and Health Survey (RAHS), 2001-2004, a 3-year cohort of 1500 older adults, and Health and Retirement Study (HRS), 1992-current, a 25-year cohort of 37 000+ older adults. As all these longitudinal cohort studies have recruited a national sample, the results are generalizable to the US population. The findings are robust and hold independent of setting, cohort, age group, psychosocial determinants, and health outcome. Other researchers have also reported similar findings^55-81^ (see Table 2).

**Table 2:** Findings by Other Researchers on the Black-White Differences in the Effects of Psychosocial Factors on Health

**Author**	**Data set**	**Longitudinal**	**Follow Up**	**Gender**	**Predictor**	**Outcome**
Kessler et al^55^	MIDUS	-	-	Both	Discrimination	Mental health
Geronimus et al^56^	-	-	-	Both	Poverty and urban stressors	Telomere length
Ferraro and Kelley-Moore^57^	NHANES	+	20 Years	Both	SRH	All-cause mortality
Zajacova and Dowd^58^	NHANES	-	-	Both	SRH	All-cause mortality
Fuller-Rowell et al^59^	-	-	-	Both	Discrimination	Diurnal cortisol rhythm
Fuller-Rowell et al^60^	-	-	-	Both	Education	Inflammation
Miller and Korenma^61^	NLSY	-	-	Both	Poverty	Stunting (low height-for-age) and wasting (low weight-for-height)
Allen et al^62^	HANDLS	-	-	Both	Urban food-insecurity	Diet quality
Barnes et al^63^	CHAP	+	5 Years	Both	Discrimination	Mortality
Tang et al^64^					APOE-3	Alzheimer disease
Rajan et al^65^	CNDS	+	7 Years	Both	Depression	Disability
Everson-Rose et al^66^	CHAP	+	7 Years	Both	Neighborhood SES	Hostility
Koebnick et al^67^	-	+	-	Both	Obesity	Asthma
Kubzansky et al^68^	MSSA	-	-	Both	SES	Distress
Gavin et al^69^	CPES	-	-	Female	Obesity	Depression
Calle et al^70^	CPS	+	14 Years	Both	Obesity	All-cause mortality
Stevens et al^71^	CPS	+	14 Years	Both	Obesity	All-cause mortality
Hogue et al^72^	NIMS	+	28 Days	Both	Birth weight	Infant mortality
Cené et al^73^	-	-	-	Both	Subjective social status	Physical and mental health
Ebong et al^74^	-	-	-	Both	Mood	Insomnia
Shaw and Krause^75^	ACL	-	-	Both	Education	Feelings of control
Plotnick et al^76^	NLSU	+	5 Years	Female	Child support	Teenage premarital childbearing
Gardener et al^77^	NMS		-	Both	Mediterranean diet	Carotid atherosclerosis
Katzmarzyk et al^78^	PCLS	+	8 Years	Both	BMI	Mortality
Anderson et al^79^	-	-	-	Both	Pain	Medication adherence
Maynard et al^80^	-	-	-	Both	Income	Obesity and myocardial infarction
Samuel et al^81^	-	-	-	Both	Community problems and resources	Systolic and diastolic blood pressure

Abbreviations: SRH‏, Self-rated Health; BMI, body mass index; ACL, Americans’ Changing Lives; SES, socio-economic status; CPS, Cancer Prevention Study II; CHAP, Chicago Health and Aging Project; CNDS, Chicago Neighborhood and Disability Study; CPES, Comprehensive Psychiatric Epidemiology Surveys; MSSA, MacArthur Studies of Successful Aging; NHANES, National Health and Nutrition Examination Survey; NIMS, National Infant Mortality Surveillance; NLSY, National Longitudinal Survey of Youth; NMS, Northern Manhattan Study; PCLS, Pennington Center Longitudinal Study.

To give a few examples of our findings, high education credentials failed to reduce the risk of physical inactivity,^44^ obesity,^44^ depressive symptoms,^45^ and suicidal ideation^46^ among Blacks. In a paper, among Black men, high educational attainment was predictive of an increase in depressive symptoms over time.^45^ Among Black women, high educational attainment was associated with high suicidal ideation.^46^

## Similar Findings in the Literature

Findings mentioned above (Table 1) have been supported by other researchers (Table 2). In a recent paper published in Social Sciences and Medicine, Malat, Mayorga-Gallob, and Williams discussed the issue.^82^ They attributed the larger effects of social and psychological factors in Whites to their Whiteness (social privilege).^82^ Williams, Kessler, Neighbors, and others have emphasized the need for systematically testing potential interactions between race and SES on health.^83,84^ Mehta has shown how behavioral risk factors interact with sociodemographic characteristics on risk of mortality.^85^ Kaufman, however, has discussed that due to potential biases such as residual confounding, it is always difficult to decompose the health effects of race from SES.^86^ Farmer and Ferraro documented largest racial gap in self-rated health at the higher levels of SES, supporting Blacks’ “diminishing returns.” Their paper showed that as education levels increases, Blacks do not gain as much self-rated health as their White counterparts.^13^ Similarly, Fuller-Rowell and colleagues found a weaker health effect of educational attainment for Blacks than for Whites.^59^ Brown et al found that eliminating gap in childhood SES, adult social and economic resources, and health behaviors do not fully eliminate racial-ethnic disparities in health trajectories, suggesting that the mechanism generating health disparities is more than differential exposures to resources.^87^

Under the same family income, Black households have a lower rate of wealth production, which has direct and indirect health implications. Compared to Whites, Black households more commonly rely on several wage earners to contribute to the total household income.^88^ Middle class Blacks are more likely than their White counterparts to be recent and tenuous in that class status.^89^ College-educated Blacks are several times more likely than their White peers to be unemployed.^90^ The purchasing power at a given level of income varies by race, as Blacks are paying higher prices than Whites for a broad range of goods and services, including food and housing.^91^ If employed, Blacks are more likely than Whites to be exposed to occupational hazards and carcinogens, even after adjusting for job experience and education.^83^

Blacks also have higher levels of goal-striving stress, John Henryism (JH), and other types of effortful coping strategies for upward social mobility. These coping strategies, however, come with psychological and physiological costs.^92-94^ Although most of the literature on health damage due to JH is limited to mental distress and depression,^95^ health disadvantage associated with JH may go beyond a psychological cost,^96,97^ particularly when high JH co-exists with low resources (SES) and social support.^92^ JH is reported to be associated with high cardiovascular risk.^98^ JH may be a resource or a health hazard,^98^ depending on the outcome and availability of other risks and resources.^95^

## What Do These Findings Mean?

According to our findings, the protective effects of psychosocial resources (eg, education, employment, and neighborhood) on health should not be considered equal between Whites and Blacks.^32,44,45^ The impact of psychosocial resources on health outcomes are conditional to factors such as poverty, residential segregation, and structural racism.^99-102^ The weaker effects of high SES for Blacks could be due to differential SES at early childhood in White and Black families. As Warner and Hayward,^103^ and Colen^104^ have argued, unequal SES at childhood may be a reason for the non-equivalence of SES effects between Whites and Blacks during adulthood. Thus, public and health policies should go beyond equalizing access to resources and additionally eliminate structural barriers that Blacks face.

According to the Blacks’ diminishing returns, education and employment have weaker effects on health of Blacks in comparison to Whites.^105^ Smaller health gains from education and employment among Blacks may be in part due to the racial wage gap in the United States labor market.^106-108^ Blacks and Whites enter different types of occupations.^109^ When employed, Blacks are paid considerably less than Whites, particularly in higher levels of education.^110^ In 2006, Black men with a master’s degree earned $27 000 less than White men with the same credentials.^111^ As Marmot has argued, although the availability of socio-economic resources is also important, what social groups can do with those resources is even more important.^110^

Increasing access to education and employment alone is not enough to eliminate racial health disparities in the United States. What is also needed is parity in wages and quality of education. Interestingly, such Black–White differences do not hold for health gains associated with income.^32^ That is income similarly reduces risk of mortality for Blacks and Whites. This finding emphasizes the importance of increasing the minimum wage and reducing the racial wage gap in the United States. Hiring and housing practices (ie, zip code discrimination; discrimination by banks in the maintenance of homes in majority Black neighborhoods) that constrain the ability of Blacks to equitably compete with Whites should also be rigorously addressed.^111^ Without improving the quality of education in majority Black schools that are limited in educational resources,^112^ and without increasing the minimum wage for Blacks, education and employment will continue to provide diminished health protection for Blacks.

## Racism Is a Multilevel System and Needs Multilevel Policy Solutions

In their 2011 *Du Bois Rev,* Gee and Ford argued that the main origin of health disparities across social groups is structural, rather than individual, phenomena. They argued that various aspects of structural racism such as social segregation, immigration policy, and intergenerational effects are in charge of maintaining health disparities. As a result, policies should attack various dimensions of structural racism as fundamental causes of health disparities.^113^

Barbara Reskin has helped us better understand the types of policies that are needed to undo racism in the United States. Drawing on a systems perspective, she has defined racism in the United States as a discrimination system that constantly generates racial disparities across multiple domains (eg, residential location, schooling, employment, health, housing, credit, and justice). Policy solutions should consider that domains are reciprocally related and comprise an integrated system. She argues that appropriate response should include implementing interventions to operate simultaneously across subsystems, and directly challenging the processes through which the emergent discrimination strengthens the subsystems.^114^

Williams and Mohammed defined racism as a multi-level system embedded in American society.^101^ Authors explained that racism adversely affects the health of non-dominant racial populations in three levels. First, through a number of policies and procedures, institutional racism has systematically reduced access of minorities to housing, neighborhood and educational quality, employment opportunities, and other desirable resources in society. Second, cultural racism operates through stereotype threat and internalized racism. Both at the societal and individual level, it generates culture and a policy environment that is hostile to egalitarian policies and triggers negative stereotypes and discrimination that are detrimental to health and foster health-damaging psychological and behavioral responses. Third, racial discrimination functions as a unique psychosocial stressor in the interpersonal and personal levels.^101^

As racial disparities are generated by a multi-level system, the responses should also target a wide range of policies that operate in those systems. First, in response to the institutional racism, policies are needed that improve neighborhood and educational quality and enhance access to additional income, employment opportunities and other desirable resources. Second, to undo disparities due to cultural racism, policies and interventions are needed at the societal and individual levels. Finally, policies are needed to maximize the health-enhancing capacities of medical care, address the social factors that initiate and sustain risk behaviors and empower individuals and communities to take control of their lives and health.^115^

Geronimus and colleagues suggest that health disparities are due to structurally-rooted biopsychosocial processes. They have coined the term Jedi Public Health (JPH) “which focuses on changing features of settings in everyday life, rather than individuals, to promote population health equity, a high priority, yet, elusive national public health objective.”^116^ They called both for an expansion and a re-orienting efforts to eliminate population health inequities. Based on their framework, there is a need for policies and interventions that remove and replace discrediting cues in everyday settings. Such policies will disrupt the repeated physiological stress process activation that fuels population health inequities.^116^

Initial advantage (eg, economic resources, health status, and cognitive ability) leads to cumulative differences that widen pre-existing gaps.^117^ For example, according to the cumulative advantage theory in the area of child development, initial advantage leads to further cumulative advantage and initial disadvantage being accentuated over time.^118^

## Widening the Gap Has Happened Before and May Happen Again

In fact, the United States may experience a widening of racial health disparities if disproportionate gains of majority and minority as well as high and low SES groups are continued. Williams and Collins have provided a historical review regarding how the gap may increase for mortality.^83^ Authors showed that a decline in Black economic well-being and an increase in Black-White inequality resulted in worsening Black health across a number of health status indicators. For instance, the Black-White gap in life expectancy widened, between 1980 and 1991, from 6.9 years to 8.3 years for males and from 5.6 years to 5.8 years for females.^83^ As explained by Williams and Collins,^83^ for every year between 1985 and 1989, the life expectancy for both African American men and women declined from the 1984 level.^119^ A slower rate of decline among Blacks than Whites for heart disease was the chief contributor to the widening racial gap in life expectancy in the past decades.^83,120^ Williams and Collins further explain that the age-adjusted Blacks to Whites death ratio was greater in 1991 than in 1980, and the annual number of excess deaths in Blacks compared to Whites showed a 6000 increase, from 60 000 to 66 000, from 1980 to 1991. During this period, the reason behind the widening gap was that the overall age-adjusted death rate decreased more rapidly for Whites than for Blacks.^83^ Freeman showed that, in the same period (1960-1980), a steady decline could be observed in national mortality; however, there was zero gain in life expectancy for Blacks in Harlem over this 20-year period.^83^

The racial gap in health worsens when the economic gap widens. In 1978, Black households received 58% of what Whites earned, but Blacks made far less compared to Whites during the 1980s. In parallel to the widening of economic gap, racial disparities in health also widened across a wide range of health indicators.^121^ For example, from 1984 to 1989, a consistent increase was observed in the life expectancy of Whites; however, the life expectancy of Blacks declined at this time.^83^

Other researchershave also described the failure of narrowing mortality disparities due to SES.^122-124^ Duleep showed that socioeconomic differences in mortality in the United States did not decline from 1960 to 1970 for men aged 25-65 years old.^124^ Feldman and colleagues found that the protective effect of education on mortality increased substantially between 1960 and 1984 for White men, but not for Black men.^123^ Pappas and colleagues compared mortality data from 1960 to 1986 and found evidence for an increase in SES disparity over that period.^125^ Wagener and Shatzkin^126^ showed that from 1969 to 1989, breast cancer mortality declined for women in high SES counties in the United States but increased for women in low SES counties.Finally, the gap in infant mortality rates for White and Black babies widened for each sex between 1980 and 1991. At the same time period, rates of both preterm delivery and low birth weight remained stable for White women, but have been increasing among Blacks.^127^ Castro showed a differential widening in the rates of sexually transmitted diseases between Blacks and Whites.^128^ These studies show that: (1) the historical widening of a racial gap has occurred and may occur again, (2) the widening of a racial gap may occur when the health of Whites and high SES groups improves with a faster pace than other groups, (3) the racial gap in health follows an increase in economic disparities, and (4) the possibilities of widening the racial gap are not specific to a single health outcome, as they spill over to multiple health domains.^83^

## Recommended Policy Solutions

Given the existing unequal gain of equal resources, policies that merely focus on the equal distribution of resources and ignore the differential distribution of barriers across groups may have the unintended effect of exacerbating the existing racial health inequities rather than reducing them. Despite their good will, employment and educational initiatives that do not account for deeply rooted structural inequalities that Blacks face may do little to reduce the racial health disparity gap in the United States. Universal investments that equalize access and ignore the structural barriers which hold Blacks behind from translating those resources to gains have the risk of widening the racial health gap, given the higher readiness of Whites to absorb such resources.

Policies and programs should be tailored to the specific needs of Blacks. Policies should specifically address multilevel structural barriers and constraints that limit Blacks’ ability to translate their available social resources and psychological assets into health gains. Socioeconomic barriers that are prevalent in Black communities should be considered, especially if the highest effects are expected for any new social and public policies that aim to reduce racial health disparities.

Racial segregation, for instance, operates as a structural and contextual barrier for many Blacks today. Discriminatory mortgage and loan policies which include higher bars and thresholds that Blacks should meet to qualify for loans in addition to higher interest rates and higher down payment for Blacks are still in practice,^129^ despite all the existing anti-discriminatory laws. Affirmative action^130^ policies may need to be reevaluated considering these findings on the diminished gains of equal resources among Blacks. Authors acknowledge that it is much easier to point out a problem. As a society, we all need to challenge the political system to approve appropriate alleviative policies.^131^

## Our Work Supports Previous Arguments

Our findings lend empirical support for the argument by Ceci and Papierno that the “Haves” always gain more than the “Have-Nots” from universal interventions. They explained that the disparities in gain which cumulates over time is a potential source of widening the disparities.^132^ They mentioned that several interventions, across different domains, have the unintended effect of widening pre-existing gaps between disadvantaged and advantaged populations, if such interventions are made available to all populations, regardless of their social and economic disadvantage status. Policy-makers should be aware of the gap-widening potential of such universal interventions and policies. Given the political and economic climate, many of the interventions will elevate the socially and economically advantaged populations to a greater degree than the disadvantaged group — certain policies may inadvertently widen the existing gap.”^132^

In 2016, Williams and Purdie-Vaughns outlined the challenge that some of the interventions that have the potential to improve health at the population level can widen social inequalities in health.^133^ They recommended that policy-makers should consider the significance of race/ethnicity in designing and developing good policies to inequalities and disparities. They also emphasized the existing need to develop a scientific research agenda to identify the distinction between the policies that reduce and those who widen the existing racial/ethnic health disparities.^133^

In 2013, Lorenc and colleagues reviewed interventions generating inequalities (IGIs),^134^ defined as effective public health interventions that increase inequalities by disproportionately benefiting less disadvantaged groups. Still, less is known about which types of interventions are likely to widen the gap, and which can reduce or eliminate the inequalities. Media campaigns; and workplace smoking bans are IGIs, however, structural workplace interventions; provision of resources; and fiscal interventions, such as tobacco pricing may reduce the gap.^134^

## Need for Further Research

Although our review clearly shows that with income being the exception, economic resources and psychological assets better protect Whites than Blacks. There is a need for research in this area that would help us better understand these differential effects.^135^ For instance, it is unclear whether upward social mobility has more social costs for Blacks than Whites.^136^ Despite Blacks have smaller health gains than Whites from most “psychological assets,” this is not the case with religious involvement and social support. Regarding religious involvement and spirituality, Blacks both report higher levels and experience greater health benefits from each unit of them. Using national data, Hummer and colleagues have shown, that high levels of religious attendance is associated with a 7 year gain in life expectancy for Whites but a 13 year gain for Blacks.^137,138^ Keyes shows that Blacks, despite all of their stress and adversity have higher levels of “flourishing” than Whites (ie, high levels of psychological well-being and low rates of mental illness).^139,140^ In 1978, Kessler showed that although Blacks and low SES persons report higher levels of stressful life events than their White and high SES counterparts, a given stressor may have more negative effects on the health of Whites and high SES persons than on their more socially disadvantaged peers.^141^ Krause,^142-144^ Assari,^145^ and others^146^ have also shown that religion involvement better promotes health of Blacks than Whites. Lincoln has also shown that social support better protects Blacks than Whites.^147^

## Conclusion

To conclude, equal resources result in unequal health gains for Whites and Blacks in the United States. Policies should not merely focus on equalizing the distribution of resources; policies should also target the differential distribution of barriers across groups.

## Ethical issues

Not applicable.

## Competing interests

Author declares that he has no competing interests.

## Author’s contribution

SA is the single author of the paper.

## Funding

Shervin Assari is supported by the Heinz C. Prechter Bipolar Research Fund and the Richard Tam Foundation at the University of Michigan Depression Center, Ann Arbor, MI, USA.
